# A broad-spectrum synthetic antibiotic that does not evoke bacterial resistance

**DOI:** 10.1016/j.ebiom.2023.104461

**Published:** 2023-02-15

**Authors:** Douglas M. Heithoff, Scott P. Mahan, Lucien Barnes V, Semen A. Leyn, Cyril X. George, Jaime E. Zlamal, Jakkarin Limwongyut, Guillermo C. Bazan, Jeffrey C. Fried, Lynn N. Fitzgibbons, John K. House, Charles E. Samuel, Andrei L. Osterman, David A. Low, Michael J. Mahan

**Affiliations:** a Department of Molecular, Cellular, and Developmental Biology, University of California, Santa Barbara, CA, 93106, USA; b Institute for Collaborative Biotechnologies, University of California, Santa Barbara, CA, 93106, USA; c Department of Medical Microbiology and Immunology, School of Medicine, University of California, Davis, CA, 95616, USA; d Infectious and Inflammatory Diseases Research Center, Sanford Burnham Prebys Medical Discovery Institute, La Jolla, CA, 92037, USA; e Center for Polymers and Organic Solids, Department of Chemistry and Biochemistry, University of California, Santa Barbara, CA, 93106, USA; f Department of Chemistry, National University of Singapore, 117543, Singapore; g Department of Medical Education, Santa Barbara Cottage Hospital, Santa Barbara, CA, 93105, USA; h Department of Pulmonary and Critical Care Medicine, Santa Barbara Cottage Hospital, Santa Barbara, CA, 93105, USA; i Division of Infectious Diseases, Santa Barbara Cottage Hospital, Santa Barbara, CA, 93105, USA; j Faculty of Science, Sydney School of Veterinary Science, The University of Sydney, Camden, New South Wales, 2570, Australia

**Keywords:** Antibiotics, Antimicrobial resistance, Multidrug-resistant pathogens, Conjugated oligoelectrolytes, COE

## Abstract

**Background:**

Antimicrobial resistance (AMR) poses a critical threat to public health and disproportionately affects the health and well-being of persons in low-income and middle-income countries. Our aim was to identify synthetic antimicrobials termed conjugated oligoelectrolytes (COEs) that effectively treated AMR infections and whose structures could be readily modified to address current and anticipated patient needs.

**Methods:**

Fifteen chemical variants were synthesized that contain specific alterations to the COE modular structure, and each variant was evaluated for broad-spectrum antibacterial activity and for *in vitro* cytotoxicity in cultured mammalian cells. Antibiotic efficacy was analyzed in murine models of sepsis; *in vivo* toxicity was evaluated via a blinded study of mouse clinical signs as an outcome of drug treatment.

**Findings:**

We identified a compound, COE2-2hexyl, that displayed broad-spectrum antibacterial activity. This compound cured mice infected with clinical bacterial isolates derived from patients with refractory bacteremia and did not evoke bacterial resistance. COE2-2hexyl has specific effects on multiple membrane-associated functions (e.g., septation, motility, ATP synthesis, respiration, membrane permeability to small molecules) that may act together to negate bacterial cell viability and the evolution of drug-resistance. Disruption of these bacterial properties may occur through alteration of critical protein–protein or protein-lipid membrane interfaces—a mechanism of action distinct from many membrane disrupting antimicrobials or detergents that destabilize membranes to induce bacterial cell lysis.

**Interpretation:**

The ease of molecular design, synthesis and modular nature of COEs offer many advantages over conventional antimicrobials, making synthesis simple, scalable and affordable. These COE features enable the construction of a spectrum of compounds with the potential for development as a new versatile therapy for an imminent global health crisis.

**Funding:**

10.13039/100000183U.S. Army Research Office, 10.13039/100000060National Institute of Allergy and Infectious Diseases, and 10.13039/100000050National Heart, Lung, and Blood Institute.

**Research in context**

**Evidence before this study**

Antimicrobial resistance (AMR) poses a critical threat to public health, disease management and global healthcare practices, with the highest burden in low-resource settings. Of particular concern are multidrug-resistant pathogens that are resistant to all, or nearly all, available antibiotics. Despite the scale and urgency, few promising drug candidates are currently in the clinical pipeline to address current and anticipated patient needs. Although multiple new compounds that function via traditional mechanisms show promise against AMR bacteria, they often are vulnerable to the same resistance mechanisms; e.g., drug efflux for synthetic fluoroquinolone derivatives. Recent discoveries have defined a promising path towards the development of antimicrobials that are not prone to antimicrobial resistance, including the natural product, teixobactin; and Irresistin, identified by small molecule library screening of unique compounds. Despite these significant advancements, considerable hurdles remain including the complexity of lead-candidate synthesis, modification, scalability and toxicity.

**Added value of this study**

Conjugated oligoelectrolytes (COEs) are a class of small synthetic molecules that were designed to insert into bacterial membranes and function as electron transporters, but some were found to inhibit bacterial growth in culture. By screening a diverse array of COEs for antibacterial activity, we identified a specific COE, COE2-2hexyl, that had broad-spectrum activity. Notably, this compound cured mice infected with pathogens derived from patients with refractory bacteremia, and did not evoke bacterial resistance. COE2-2hexyl had specific effects on multiple membrane-associated functions that may act together to disrupt bacterial cell viability and the evolution of drug-resistance through a mechanism of action distinct from most membrane disrupting antimicrobials or detergents which destabilize membranes to induce cell lysis.

**Implications of all the available evidence**

The diversity and ease of COE design and chemical synthesis have the potential to establish a new standard for drug design and personalized antibiotic treatment. These COE features enable the construction of a spectrum of compounds with the potential as a new versatile therapy for the emergence and rapid global spread of pathogens that are resistant to all, or nearly all, existing antimicrobial medicines.

## Introduction

The World Health Organization (WHO) identified antimicrobial resistance among the major threats to global health, food security and economic stability,^1^ attributing to 1.27 million deaths annually with the highest burden in low- and middle-income countries.^2^ This public health crisis is predicted to worsen due to climate change—a force-multiplier for the spread of infectious disease and antimicrobial resistance.^3^ Of particular concern are CDC urgent/WHO critical priority pathogens that are resistant to all—or nearly all—available antibiotics.^4^^,^^5^ These include carbapenem-resistant *Acinetobacter, Enterobacterales*, and *Pseudomonas aeruginosa* (CRAB, CRE, CRPA); *Clostridioides difficile*; drug-resistant *Neisseria gonorrhoeae*; and extended spectrum β-lactamase producing Enterobacteriaceae (ESBL). Despite the imminent public health threat, few promising drug candidates are currently in the clinical pipeline^6^^,^^7^ due to the high costs of drug development and the risk that a new antibiotic becomes ineffective due to bacterial resistance or is reserved as a drug of last resort.^8^^,^^9^ Other factors include the diminished incentives for pharmaceutical research and development for diseases that require relatively short courses of treatment (infectious diseases) compared to blockbuster drugs for pervasive diseases (cancer, cardiovascular diseases, hyperlipidemia and immune disorders).^10^^,^^11^

Product profiles of new antimicrobials that do not evoke bacterial resistance are critical to both clinical and marketplace needs.^12^ Recent discoveries have defined a promising path towards developing antibiotics that do not evoke bacterial resistance, including the natural product teixobactin that specifically targets Gram-positive bacteria, and Irresistin, a broad-spectrum antibiotic identified by small molecule library screening of unique compounds.^13^^,^^14^ However, significant hurdles remain including complexity of synthesis, modification, scalability and toxicity of lead candidates.

**Fig. 1:**
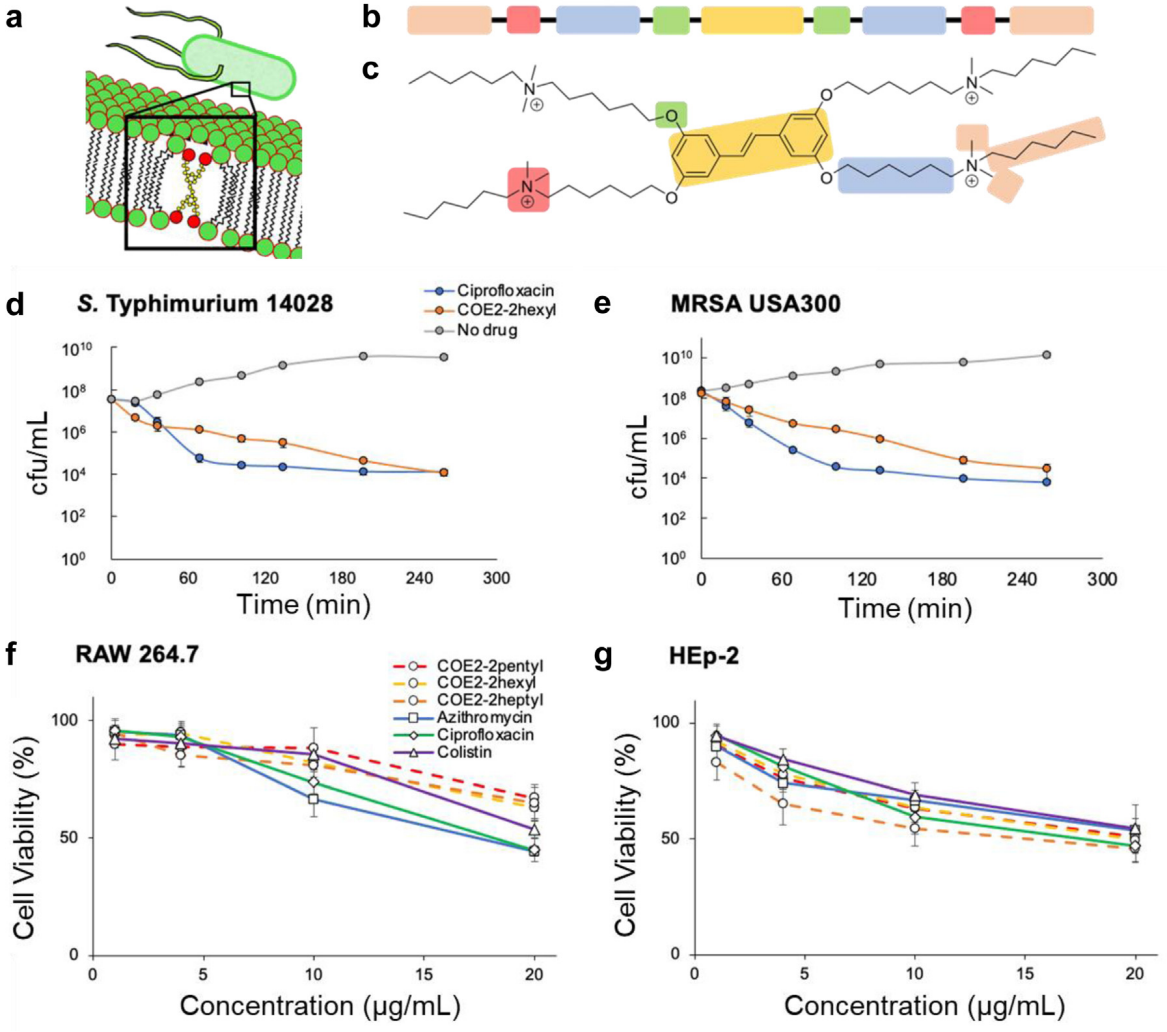
**Conjugated oligoelectrolytes (COEs) and comparative bactericidal activity and mammalian cell cytotoxicity. a,** COEs share a modular structure that spontaneously integrates into the bacterial membrane. **b** and **c,** COE structural modules are depicted by coloured boxes. The intercalation into phospholipid bilayers is driven by the hydrophobic centre and the terminal ionic functionalities, consisting of the conjugated aromatic core (gold module) and hydrocarbon pendants (blue module), which resemble the fatty acid centre of the bilayer. Additionally, the cationic end groups (red module) and terminal acyl chains (pink module) interact via coulombic and hydrophobic interactions with the membrane surface functionalities; specific example: COE2-2hexyl. Bactericidal activity. Exponential-phase cultures (∼10^8^ cells) of either **d,***S.* Typhimurium 14028 or **e,** CA-MRSA USA300 were incubated with 10 × MIC of either COE2-2hexyl (20 μg/mL; 10 μg/mL, respectively) or ciprofloxacin (0.156 μg/mL; 5 μg/mL, respectively) for 4 h, and viability was enumerated by direct colony count (n ≥ 3, SEM). Mammalian cell cytotoxicity. COEs at the designated concentration (1–20 μg/mL) were incubated for 18 h with **f,** murine macrophage (RAW 264.7) and **g,** human epithelial cell lines (HEp-2). Mammalian cell cytotoxicity was determined by the trypan blue vital stain exclusion method,^15^ and the unstained viable cells were counted using a hemocytometer (n = 6, SD).

Conjugated oligoelectrolytes (COEs) are a class of small amphiphilic molecules that share a modular structure that can spontaneously interact with lipid bilayers (Fig. 1a).^16^^,^^17^ The ease of molecular design and synthesis allows the construction of a spectrum of bacterial interfacing synthetic compounds that can be readily modified to alter membrane affinity and other properties (solubility, charge, stability).^18^^,^^19^ COE intercalation into phospholipid bilayers is driven by the hydrophobic centre and the terminal ionic functionalities, consisting of the conjugated aromatic core and hydrocarbon pendants. These pendant moieties resemble the fatty acid centre of the bilayer, while the cationic end groups and terminal acyl chains interact via coulombic and hydrophobic interactions with the membrane surface functionalities (Fig. 1b and c). Although COEs were initially designed to insert into bacterial membranes and function as electron transporters, some were found to inhibit bacterial growth in culture for a limited number of pathogens.^20^^,^^21^ Such findings launched an effort to synthesize and screen a diverse array of COE chemical variants (altered length of the conjugated aromatic backbone, distribution of ionic groups, and hydrophobic substitutions) for antibacterial activity against 17 clinically relevant Gram-negative and Gram-positive pathogens. Here we report on a specific COE, COE2-2hexyl, that exhibited broad-spectrum activity, effectively treated mice infected with multidrug-resistant (MDR) bacteria and was not prone to bacterial resistance.

## Methods

### Synthesis and characterization of conjugated oligoelectrolytes

COE syntheses and product characterizations were as previously described (detailed experimental methods, synthetic schemes, yields and NMR spectra).^19^, ^20^, ^21^, ^22^, ^23^ Briefly, the alkylation steps were performed by Williamson ether synthesis with a carbonate base. The COE conjugated backbones were derived via Horner−Wadsworth−Emmons or McMurry coupling reactions. The specific COEs were obtained after quaternization of the terminal alkyl halide groups with excess trimethylamine or other amines. Intermediates and COE products were purified using multiple strategies (i.e., liquid−liquid extraction, column chromatography, precipitation, solvent removal under vacuum, etc.) and subsequently characterized by NMR or mass spectroscopy. The maximum solubility of COE2-2hexyl is 400 μg/mL in sterile H_2_O. COE2-2hexyl MIC testing was performed on at least three independent batch syntheses.

### Bacterial strains and culture conditions

Gram-negative bacterial isolates included: *A. baumannii* ATCC 19606; *A. baumanii* ATCC 17978; *E. coli* DH5α, *E. coli* ATCC 25922, *E. coli* MG1655, *E. coli* BW25113, *E. coli* BW25113 Δ*mutL::kan*, *K. pneumoniae* ATCC 13883; CRE *K. pneumoniae* (MT3325), derived from a urinary/sepsis patient obtained from Santa Barbara Cottage Hospital (2017); *N. gonorrhoeae* ATCC 700825; *N. gonorrhoeae* ATCC 49226; *S. flexneri* ATCC 29903; *P. aeruginosa* ATCC 10145; *S. enterica* serovar Typhimurium ATCC 14028; *Y. pseudotuberculosis* (YPIII). Gram-positive clinical isolates included methicillin-resistant (MRSA) and -sensitive (MSSA) *S. aureus*: CA-MRSA USA300, MSSA Newman and 3 isolates derived from sepsis patients obtained from Santa Barbara Cottage Hospital (2016) termed MRSA Blood (MT3302); MRSA Wound (MT3315); MSSA Blood (MT3305).^24^ *S. pneumoniae* clinical isolates included D39 (ser. 2) and Daw 1 (ser. 6).

Bacterial strains were grown as previously described.^25^ Briefly, Gram-negative bacteria were isolated after overnight growth on Luria–Bertani (LB) agar and incubated at 37 °C in ambient air or, for *Yersinia*, 48 h at room temperature. *N. gonorrhoeae* were isolated on Chocolate agar (Becton Dickinson) for 48 h at 37 °C in a 5% CO_2_ incubator. Gram-positive *S. aureus* strains were isolated on Tryptic Soy Broth (TSB) agar and incubated at 37 °C in ambient air. *S. pneumoniae* strains were grown overnight on Columbia CNA agar with 5% sheep blood (Becton Dickinson) and grown in Todd-Hewitt Broth (THB) supplemented with 2% yeast extract incubated at 37 °C in a 5% CO_2_ incubator.

### MIC assays

COE structural variants were evaluated for MIC against a collection of nine clinical bacterial isolates via broth microdilution (n ≥ 9).^26^, ^27^, ^28^ Gram-negative bacteria: *E. coli* ATCC 25922 (MT3277); *K. pneumoniae* ATCC 13883 (MT1947); *P. aeruginosa* ATCC 10145 (MT1945); and *S*. Typhimurium 14028; Gram-positive bacteria: MSSA Newman; MSSA blood isolate (MT3305); CA-MRSA USA300; MRSA blood isolate (MT3302); and MRSA wound isolate (MT3315). Standard AST medium is Mueller-Hinton Broth (MHB) supplemented with CaCl_2_ and MgCl_2_ to make cation-adjusted MHB (Ca-MHB).^26^ Unless otherwise specified, bacteria were grown overnight at 37 °C in Ca-MHB broth in ambient air. *Yersinia* was cultured at 28 °C. *N. gonorrhea* was grown in modified Chocolate broth^29^ incubated at 37 °C for 20 h in a 5% CO_2_ incubator. *S. aureus* MIC assays were done by direct inoculation: five to seven *S. aureus* colonies from TSB agar were used to inoculate 1 mL Ca-MHB. *S. pneumoniae* was grown overnight on Columbia CNA agar with 5% sheep blood, and five colonies were inoculated into 0.5 mL Ca-MHB supplemented with 5% lysed horse blood (Lampire Biological Laboratories), and incubated 4 h at 37 °C in a 5% CO_2_ incubator.

### Cell culture

The murine macrophage RAW 264.7 (ATCC TIB-71) and the human epithelial HEp-2 (ATCC CCL-23) cell lines were obtained from the American Type Culture Collection, Rockville, MD, and maintained in minimum essential medium (MEM) supplemented with l-glutamine and 10% heat-inactivated bovine growth-supplemented calf serum (HyClone Laboratories, Logan, UT). Cells were grown in a humidified atmosphere of 5% carbon dioxide and 95% air at 37 °C in 75-cm^2^ plastic flasks (Corning Glass Works, Corning, NY). Cultured cells were harvested by scraping with a rubber policeman and plated at a density of 5 × 10^4^ to 1 × 10^5^ cells/mL in 1 mL of supplemented MEM in 24-well dishes (Corning) and grown for 24 h to approximately 80–90% confluence (1 × 10^5^–2 × 10^5^ cells/well) (adapted from ^30^).

### COE cytotoxicity in cultured mammalian cells

*In vitro* cytotoxicity was determined by the trypan blue vital stain exclusion method.^15^ COEs or antibiotics were added to cultured murine macrophage (RAW 264.7) or human epithelial (HEp-2) 80–90% confluent monolayers in 24-well cell culture plates (Corning) at 0, 1, 4, 10 or 20 μg/mL in 1 mL cell culture media. The culture was incubated for 18 h at 37 °C in a 5% CO_2_ incubator, washed once with 1 mL PBS, and cells were harvested by scraping into 0.1 mL PBS. Cells were diluted 1:5 in PBS, and 10 μL of the diluted cells were added to an equal volume of 0.4% trypan blue, and the unstained viable and total cell number were counted using a hemocytometer. Standard deviation (SD) was determined from 6 biological replicates for each condition.

### Bactericidal activity assay

*S.* Typhimurium 14028 or CA-MRSA USA300 were grown overnight in LB or TSB respectively, diluted 1:100, and incubated at 37 °C with shaking to obtain exponential-phase cultures. ∼10^8^ cells of *S.* Typhimurium 14028 or CA-MRSA USA300 were incubated with either 10 × MIC of COE2-2hexyl (20 μg/mL; 10 μg/mL, respectively) or ciprofloxacin (0.156 μg/mL; 5 μg/mL, respectively) 4 h, and viability was enumerated by direct colony count. Standard error of the mean (SEM) was determined from ≥3 biological replicates for each condition.

### In vivo toxicity assay

Toxicity of COE2-2hexyl was assessed in mice relative to that of low- and high-dose treatment with polymyxin B (PMB). The proportion of mice developing abnormal attitude scores was compared between groups using Fisher's exact test with Bonferroni adjustment for multiple comparisons. A linear mixed model was also fitted with attitude score as the outcome variable, treatment group was evaluated as a factor and mouse as a random effect. Differences in attitude scores were determined for PMB treatment among low- and high- dose groups [i.p., 30 mg/kg/day (B.I.D) vs. 45 mg/kg/day (T.I.D.)]^31^ and mock-treated BALB/c mice (n = 10); and between the COE2-2hexyl treated (i.v., 2 mg/kg/day) (S.I.D.) and mock-treated C57BL/6 mice (n = 10). Attitude score scale: 0 = normal appearance/activity/clinical signs; 1 = slight abnormal hair coat; normal activity/clinical signs; 2 = abnormal hair coat; normal activity/clinical signs; 3 = abnormal appearance/activity/clinical signs, euthanize.

### Bacterial infections and antibiotic treatment

Bacterial infections were performed as previously described.^25^^,^^32^ Mice were i.v. infected with either MRSA (MT3302) or CRE *K. pneumoniae* (MT3325) at a dose of 1 × 10^8^ cfu (20 × LD_50_) and treated for 3 days (beginning 2 h post-infection) with a single-daily dose of COE2-2hexyl (i.v., 2 mg/kg/day; maximum soluble dose) (n = 10). Additionally, another CRE *K. pneumoniae* cohort was treated with a twice-daily dose of colistin (CST) (i.p. 30 mg/kg/day)^24^ (n = 10). Survival was scored up to day 5 and compared to infected, mock-treated animals (MRSA, n = 10; *K. pneumoniae*, n = 20). The COE2-2hexyl dose is equivalent to 28 μg/mL plasma concentration at the time of administration (28 × MIC for MRSA; 7 × MIC for *K. pneumoniae*). Unless otherwise specified, all animal experiments were carried out with 8–12-week-old C57BL/6J mice and used equal numbers of males and females. All mice in the study were provided sterile pellet food and water ad libitum.

### DiBAC_4_ and PI staining

Overnight cultures of *E. coli* MG1655 cells were diluted 100-fold in LB media, grown to log phase with shaking at 37 °C, diluted in LB to an OD_600_ = 0.2 and aliquoted (2 mL each) into polypropylene tubes. COE2-2hexyl was added at the indicated final concentrations and cells were incubated at 37 °C with shaking for the times indicated. At the end of the incubation period, cells were harvested by centrifugation (15,000 × g, 1 min, 23 °C), resuspended in an equal volume of phosphate-buffered saline, pH 7.2 (PBS), and DiBAC_4_(3) and/or PI were added at final concentrations of 0.01 μg/mL and 6.68 μM respectively, and incubated for 30 min at 37 °C. Samples were then analysed using an Accuri C6 flow cytometer (Becton Dickinson) using FL1 (533/30 nm, DiBAC_4_(3)) and FL3 (670 nm, PI) fluorophore filters. SD was determined from 3 biological replicates.

### ATP analysis

Exponentially growing *E. coli* MG1655 cells were diluted in LB medium to OD_600_ = 0.2, COE2-2hexyl was added to 2 mL cultures of these cells in polypropylene tubes and incubated with shaking for the indicated times at 37 °C. At the end of the incubation time, 100 μL samples were dispensed into 3 wells (triplicate) of an opaque black microtiter plate and 100 μL of BacTiter-Glo reagent (Promega) was added. After incubation with gentle shaking for 5 min at 23 °C, luminescence was measured using a Perkin–Elmer Wallac 1420 multilabel counter. Cultures similarly grown and processed without the addition of COE2-2hexyl served as control. In parallel, serially-diluted pure ATP preparations were subjected to the same assay and the luminescence output measured and plotted to calculate ATP levels in samples. SD was determined from 3 biological replicates.

### Oxygen consumption and protein quantification

Overnight cultures of *E. coli* MG1655 cells were diluted 1:100 with LB media and grown with shaking at 37 °C to OD_600_ = 0.6. Cultures were then diluted in LB medium to OD_600_ = 0.2, 5 mL aliquots were taken in polypropylene tubes, and COE2-2hexyl was added and incubated at 37 °C with shaking for the indicated times. Aerobic respiration was measured at 23 °C in a sealed stirred cuvette with a Clarke oxygen electrode as recommended by the manufacturer (Qubit Systems). Calibration was carried out after air was bubbled into the oxygen electrode sample chamber (100% O_2_ saturation) and after addition of a few grains of sodium sulphite (0% saturation). *E. coli* samples (3 mL) were added to the calibrated cuvette equipped with a stir bar; the top plunger was lowered, and air bubbles were expelled through the top port. Readings were taken every second and the slope was calculated by least-squares analysis.^33^ Protein quantification was carried out using the DC protein assay (Bio-Rad). Briefly, bacterial cultures (1 mL) were harvested by centrifugation (15,000 × g, 1 min, 23 °C), and cell pellets were washed 1× with 200 μL PBS. The pellets were resuspended in 100 μL 1× Laemmli buffer, heated at 100 °C for 5 min, centrifuged at 15,000 × g for 5 min and the supernatant solutions collected. Samples were analysed according to the Bio-Rad protocol measuring absorbance at 750 nm, measuring dilutions of a bovine serum albumin control solution in parallel to quantify total protein content. SD was determined from 3 biological replicates.

### Cloxacillin sensitivity

The effect of COE2-2hexyl on cloxacillin sensitivity was measured using *E. coli* DL5916 (MG1655 *acrB::EZ-Tn ΔaraBAD::spc*^*R*^) and DL5850 (*acrB*^+^*ΔaraBAD::spc*^*R*^). Serial dilutions of COE2-2hexyl were made in LB (1 mL, polypropylene tubes), 10 μL of *E. coli* (OD_600_ = 0.2) was added to each tube and tubes incubated overnight with shaking to estimate the MIC for each *E. coli* strain (in triplicate, experiment repeated 3 times). Similarly, the MIC of cloxacillin was determined for both strains, both in the absence of COE2-2hexyl and in the presence of 0.5 × MIC COE2-2hexyl (1 μg/mL). SD was determined from 4 biological replicates.

### Isolation of COE^R^ mutants by serial dilution

Three independent 10 mL cultures of bacteria were grown overnight, serially diluted 1:10 in the presence of either 0.5 × MIC or 1 × MIC COE2-2hexyl in polypropylene flasks, and incubated 20 h at 37 °C with shaking (MIC: *S.* Typhimurium 14028, 2 μg/mL; MRSA CA–USA300, 1 μg/mL). If growth was scored in the presence of 1 × MIC, the serial dilution procedure was repeated at the initial- and double-the drug concentration (1 and 2 × MIC). If no growth was scored at 1 × MIC, the 0.5 × MIC culture was used to repeat the serial dilution procedure (0.5 and 1 × MIC). If growth then occurred at 1 × MIC, the serial dilution procedure was repeated at the initial- and double-the drug concentration (1 and 2 × MIC). This procedure was repeated for 21 consecutive days.

### Isolation of COE^R^ mutants by morbidostat-based experimental evolution

The morbidostat measures the growth rates of evolving microbial populations and automatically adjusts drug concentrations to maintain a constant drug-induced inhibition.^34^^,^^35^ The hardware, software components and experimental methods and work-flow were as described.^34^ Briefly, bacterial populations were placed under increasing selective drug pressure in parallel reactors. Total genomic DNA from bacterial cell populations from each reactor were sampled and sequenced on a daily basis; and mutations and possible mechanisms of resistance were determined by variant calling bioinformatics. Experimental validation was performed by verification of identified mutations and measurement of the MIC change of representative clones.

### Ethics statement

Human subjects approval was obtained from the Institutional Human Subjects Use Committee of the University of California, Santa Barbara and the Institutional Review Board of Santa Barbara Cottage Hospital. All animal experimentation was conducted following the National Institutes of Health guidelines for housing and care of laboratory animals and performed in accordance with Institutional regulations after pertinent review and approval by the Institutional Animal Care and Use Committee at the University of California, Santa Barbara.

### Statistical analyses

Log-rank (Mantel–Cox) test was used to compare differences in survival between groups for Kaplan–Meier survival curves; significance was determined using GraphPad Prism version 9.0. *P* values of less than 0.05 were considered significant. The exact value of n, representing the number of mice, was indicated in the figure legends. Fisher's exact test and Restricted Maximum Likelihood (REML) were used to compare differences of *in vivo* toxicity utilizing RStudio^36^ and the fmsb (version 0.7.3)^37^ and lme 4 (version 1.1–29)^38^ packages.

### Role of the funding source

The funders of the study had no role in the study design, data collection, data analysis, data interpretation, or writing of the report.

## Results

### In vitro COE screen for antibacterial activity and cytotoxicity

Fifteen chemical variants were synthesized that contain specific alterations to the COE modular structure, including length of conjugated aromatic backbone, linkage type, length of hydrocarbon pendants, and distribution of cationic groups and terminal acyl chains (Supplementary Table S1; see Methods). Each structural variant was evaluated for antibacterial activity against a collection of nine bacterial clinical isolates via antibiotic susceptibility testing (AST)—defining the minimal inhibitory concentration (MIC) of each compound^26^, ^27^, ^28^—and for *in vitro* cytotoxicity relative to ciprofloxacin (CIP), a broad-spectrum fluoroquinolone antibiotic (Supplementary Table S1). Following incubation of a murine macrophage cell line (RAW 264.7) with these compounds, membrane integrity as a measure of cytotoxicity was determined by the trypan blue vital stain exclusion method.^15^ This *in vitro* efficacy/cytotoxicity screen identified a lead candidate, COE2-2hexyl, which was the only structural variant tested with antibacterial activity against Gram-negative pathogens, while exhibiting similar levels of *in vitro* cytotoxicity relative to CIP treatment (Fig. 1c; Supplementary Table S1). Although two other COEs were identified from this screen (distyrylbenzene oligoelectrolyte [DSBN] and COE2-3C), they were only effective against Gram-positive bacteria.

Expanded antibacterial activity analyses revealed that COE2-2hexyl exhibited broad antibacterial activity against all 17 clinical bacterial isolates tested (Table 1). Notably, methicillin-resistant *S. aureus* (MRSA, MT3302) and CRE *K. pneumoniae* (MT3325) were derived from sepsis patients with refractory bacteremia, whereby the CRE organism was resistant to 20/22 antibiotics determined by clinical VITEK testing (bioMerieux, Inc.) and 19/24 antibiotics determined by broth microdilution (Supplementary Table S2). COE2-2hexyl was then evaluated for bactericidal activity against *Salmonella* Typhimurium and MRSA relative to CIP treatment. Bacterial cultures were incubated with either 10 × MIC of COE2-2hexyl or CIP and bacterial cell viability was determined as a function of time. COE2-2hexyl effectively killed both *S.* Typhimurium 14028 and CA-MRSA USA300, with bactericidal activity kinetics similar to that of CIP treatment (Fig. 1d and e).

**Table 1:** COE antibacterial activity against clinical bacterial isolates.

Pathogen	Antibacterial activity MIC (μg/mL)						
	AZM		CIP		COE2-2hexyl	DSBN	COE2-3C
**Gram-negative**							
*A. baumannii* ATCC 19606	64	R	1	S	4	128	128
*E. coli* ATCC 25922	4	S	0.008	S	2	128	8
*K. pneumoniae* ATCC 13883	8	S	0.031	S	2	128	32
*K. pneumoniae* (CRE)^a^	256	R	128	R	4	256	64
*N. gonorrhea* ATCC 700825	0.031	S	0.002	S	0.5	4	0.5
*N. gonorrhea* ATCC 49226	0.063	S	0.002	S	1	16	1
*P. aeruginosa* ATCC 10145	128	R	0.125	S	8	>256	256
*S. flexneri* ATCC 29903	2	S	0.016	S	2	64	8
*S. Typhimurium* 14028	4	S	0.016	S	2	64	8
*Y. pseudotuberculosis* YPIII	8	S	0.008	S	1	128	16
**Gram-positive**							
MSSA Newman	1	S	0.25	S	1	1	1
MSSA blood isolate (MT3305)	>512	R	0.125	S	1	2	0.5
MRSA CA–USA300	128	R	0.5	S	1	1	0.5
MRSA blood isolate^a^ (MT3302)	128	R	0.5	S	1	1	0.5
MRSA wound isolate (MT3315)	1	S	16	R	1	2	1
*S. pneumoniae* D39	0.031	S	0.5	I	4	32	8
*S. pneumoniae* Daw 1	8	R	0.5	I	8	32	4

MICs and susceptibility designations were determined by broth microdilution^26^, ^27^, ^28^ (n ≥ 9).

S, susceptible; I, intermediate; R, resistant; AZM, azithromycin; CIP, ciprofloxacin; DSBN, distyrylbenzene oligoelectrolyte.

a Bacterial isolate derived from a patient refractory to antibiotic therapy.

Because COE2-2hexyl was the only compound tested that conferred broad-spectrum antibacterial activity, two chemical derivatives were synthesized, COE2-2pentyl and COE2-2heptyl, each differing from COE2-2hexyl by one hydrocarbon in the terminal acyl chain that affects hydrophobicity and interaction with the membrane bilayer.^20^ Similar to COE2-2hexyl, these derivatives conferred broad-spectrum activity against all 9 clinical bacterial isolates tested (Supplementary Table S3). They also maintained low *in vitro* cytotoxicity upon incubation with either murine macrophages (RAW 264.7) or human epithelial cells (HEp-2) relative to clinically-relevant antibiotics (azithromycin, ciprofloxacin and colistin) (Fig. 1f and g). Drug concentrations are expressed as μg/mL because MIC values observed for a given drug depend upon the bacterial species and particular isolate evaluated (Table 1; Supplementary Table S1). These data suggest that COE2-2hexyl and close synthetic derivatives define a robust structural motif for broad-spectrum antibacterial activity and low cytotoxicity in cultured mammalian cells.

### In vivo COE2-2hexyl toxicity/efficacy

**Fig. 2:**
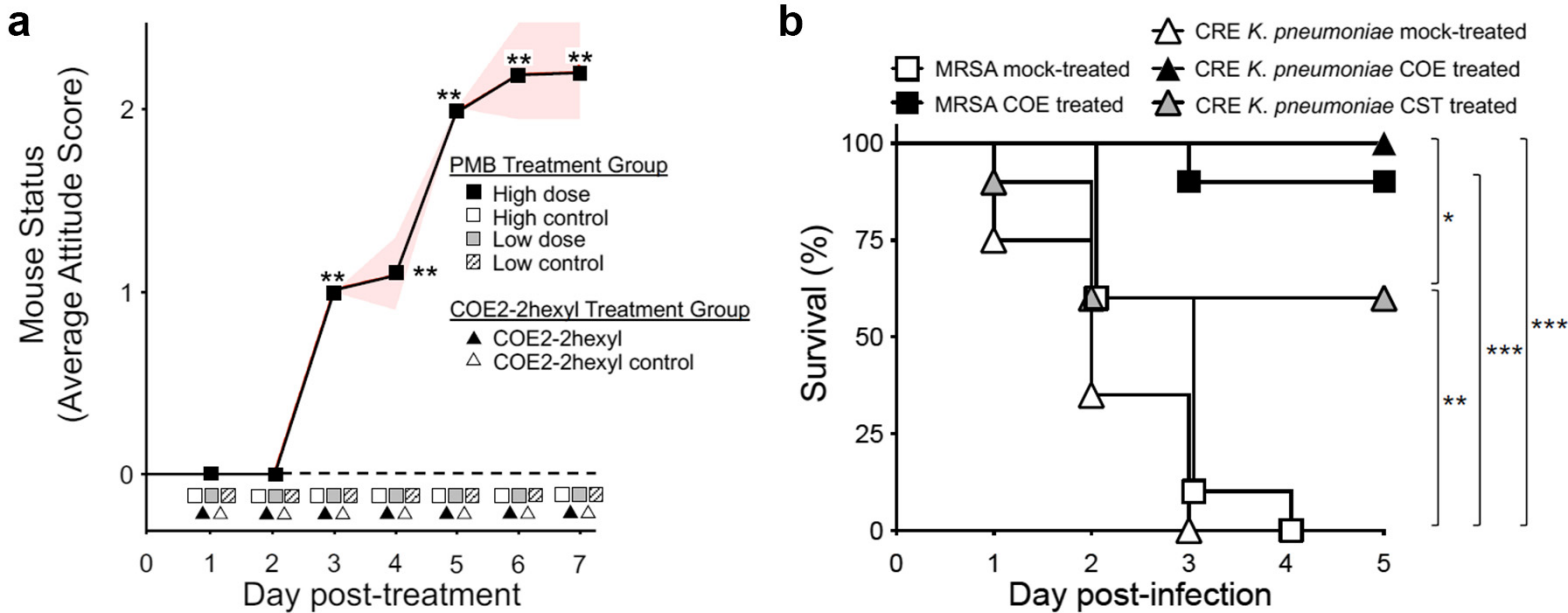
**COE2-2hexyl toxicity and efficacy in mice. a,**Toxicity of COE2-2hexyl was assessed in mice relative to that of low- and high-dose treatment with polymyxin B (PMB) via blinded-daily measurement of attitude scores as an outcome variable upon drug treatment. Differences in the proportion of mice with abnormal scores in each group and differences in attitude scores between groups were determined for PMB treatment between low- and high- dose groups (i.p., 30- vs. 45- mg/kg/day)^31^ and between mock-treated mice (n = 10); and between the COE2-2hexyl treatment group (i.v., 2 mg/kg/day, maximum soluble dose) and mock-treatment mice (n = 10). The shaded areas reflect the 95% confidence interval of the mean attitude scores. Attitude scale: 0 = normal appearance/activity/clinical signs; 1 = slight abnormal hair coat; normal activity/clinical signs; 2 = abnormal hair coat; normal activity/clinical signs; 3 = abnormal appearance/activity/clinical signs, euthanize. Values at or below hatched line = zero attitude score. **b,**Efficacy. Mice were i.v. infected with either MRSA (MT3302) or CRE *K. pneumoniae* (MT3325) at a dose of 1 × 10^8^ cfu (20 × LD_50_)^24^ and treated for 3 days (beginning 2 h post-infection) with a single-daily dose of COE2-2hexyl (i.v., 2 mg/kg/day) (n = 10). Additionally, another CRE *K. pneumoniae* cohort was treated with a twice-daily dose of colistin (CST) (i.p. 30 mg/kg/day)^24^ (n = 10). Survival was scored up to day 5 and compared to infected, mock-treated animals (MRSA, n = 10; *K. pneumoniae*, n = 20). ∗*P* < 0.05; ∗∗*P* < 0.01; ∗∗∗*P* < 0.001.

*In vivo* toxicity of COE2-2hexyl was assessed relative to polymyxin B (PMB), a cationic, cyclic polypeptide antibiotic that is cytotoxic to renal tubular cells, accumulates to high levels in the kidney, and is prone to nephrotoxicity and neurotoxicity.^39^ *In vivo* toxicity was analysed via a blinded measurement of attitude scores as an outcome of drug treatment (appearance/activity/clinical signs). A significant difference in attitude scores was observed between PMB treatment of low- and high- dose groups (i.p., 30- vs. 45- mg/kg/day)^31^ (Fig. 2a). All low dose medicated mice remained normal whereas all high dose treated mice expressed abnormal attitude scores. Pairwise comparisons of attitude scores by study day indicated the low-dose group had significantly lower attitude scores on days 3 through 7, similar to that of mock-treated mice (n = 10) (*P* = 0.004 [Fisher's exact test]). No significant difference in attitude scores was observed between COE2-2hexyl treatment (i.v., 2 mg/kg/day; maximum soluble dose) and mock-treated mice (n = 10). These data indicate that *in vivo* toxicity was not detectable via blinded measurement of attitude scores as an outcome of COE2-2hexyl treatment.

Next, COE2-2hexyl efficacy was examined *in vivo* with pathogenic isolates derived from sepsis patients resistant to conventional antibiotic therapy (Supplementary Table S2). Mice were i.v. infected with either MRSA (MT3302) or CRE *K. pneumoniae* (MT3325) at a dose of 1 × 10^8^ cfu (20 × LD_50_) and treated for 3 days with a single-daily dose of COE2-2hexyl (i.v., 2 mg/kg/day) (n = 10). Additionally, another CRE *K. pneumoniae* cohort was treated with a twice-daily dose of colistin (CST) (i.p. 30 mg/kg/day)^24^—a last-line therapy for carbapenem-resistant Gram-negative infections^40^ (n = 10). Survival was scored up to day 5 and compared to infected, mock-treated animals. COE2-2hexyl conferred high-level protection in both murine sepsis models (MRSA, 9/10 survivors; *K. pneumoniae*, 10/10 survivors) relative to infected, mock-treated animals (MRSA, 0/10 survivors; *K. pneumoniae*, 0/20 survivors) (Fig. 2b; *P* < 0.001 [log-rank (Mantel–Cox) test]). Furthermore, although CRE *K. pneumoniae* was markedly susceptible to CST upon *in vitro* testing (MIC 32-fold below the resistance breakpoint; Supplementary Table S2), it conferred significantly less protection relative to COE2-2hexyl (6/10 survivors vs. 10/10 survivors; *P* = 0.03 [log-rank (Mantel–Cox) test]) (Fig. 2b). Taken together, these data suggest that COE2-2hexyl was not intrinsically toxic *in vivo*, and effectively treated MDR pathogens in murine models of sepsis.

### Mechanistic studies of COE action

**Fig. 3:**
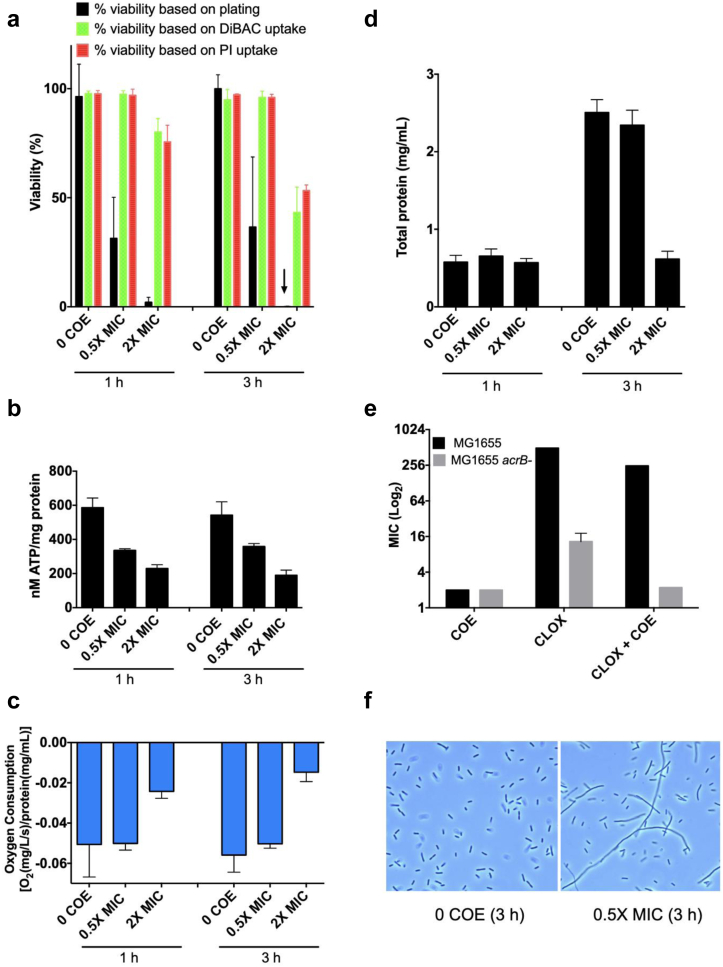
**Effects of COE2-2hexyl on membrane and cellular integrity.***E. coli* MG1655 cells were grown and treated with COE2-2hexyl and analysed as described in Methods. **a,**Viability assays. *E. coli* was treated with COE2-2hexyl at the MICs indicated for 1 h and 3 h. Viability was assessed by counting colonies after plating cell dilutions on LB medium, quantifying PI uptake, and quantifying DiBAC_4_(3) uptake (n = 3, SD), The mean % viability indicated by the arrow was 0.11%, SD = 0.14. **b,**Cellular ATP content was estimated using BacTiter-Glow reagent and total protein content was quantified using the Bio-Rad protein DC assay to calculate nM ATP/mg protein. **c,**Oxygen consumption was determined using the OX1LP dissolved oxygen package (Qubit Systems) as described in Methods (n = 3, SD). **d,**Total cellular protein was determined as described in Methods using the Bio-Rad DC protein assay (n = 3, SD). **e,**Cloxacillin treatment. *E. coli* DL5850 (*acrB*^+^) and DL5916 (*acrB*^−^) cells were treated with COE2-2hexyl (COE), cloxacillin (CLOX), or cloxacillin plus 0.5 × MIC COE (CLOX + COE) as indicated on the x-axis to determine the effect of a sub-MIC level of COE on cloxacillin resistance (n = 4, SD). **f,**Cellular morphology. *E. coli* MG1655 was incubated in the presence of 0.5 × MIC or the absence of COE for 3 h and cellular morphology was examined by phase-contrast microscopy (100×).

Fundamental physiologic analyses were performed to explore the COE mechanism of action using *E. coli* K-12 (MG1655). COE2-2hexyl treatment for 3 h with 0.5 × MIC resulted in ∼3-fold reduction in bacterial cell viability, whereas 2 × MIC resulted in a 1000-fold viability reduction (Fig. 3a). Next, we analyzed uptake of DiBAC_4_ and propidium iodide (PI) as approaches to assess membrane integrity.^41^^,^^42^ DiBAC_4_ is a membrane potential-sensitive dye used to evaluate the proton motive force (PMF), whereas PI is a membrane impermeable DNA-binding stain. Notably, neither uptake of DiBAC nor PI paralleled bacterial cell viability. At 2 × MIC, only about 20% of the cells stained positive for both dyes at 1 h, and 50% stained at 3 h based on flow cytometric analyses (see Methods). These data suggest that the effects of COE2-2hexyl on membrane permeability cannot account for the large effects on bacterial cell viability.

Quantification of cellular ATP level has been used to gauge bacterial metabolic activity and cell viability.^43^ After 1 h of incubation, COE2-2hexyl reduced ATP levels by 1.7-fold and 2.5-fold at 0.5 × MIC and 2 × MIC, respectively, while a similar reduction in ATP level was measured after 3 h (Fig. 3b). These results are in contrast with the marked decrease in bacterial cell viability. A similar disparity was observed with cellular respiration, which was reduced by only 2.1-fold at 1 h and 3.4-fold after a 3 h incubation with 2 × MIC (measured by oxygen consumption), while 0.5 × MIC no effect (Fig. 3c). Notably, 2 × MIC blocked cell protein increases between the 1 h and 3 h time points, indicating that cells did not grow over the time course; 0.5 × MIC had no effect (Fig. 3d). Taken together, these metabolic data indicate that COE-mediated alterations in ATP content and respiration did not parallel the marked effects on bacterial cell viability.

To further explore the effects of COE2-2hexyl on cell membrane-related functions, we determined if COE2-2hexyl altered the susceptibility of *E. coli* to the broad-spectrum penicillin derivative, cloxacillin. Cloxacillin is exported via the inner membrane protein AcrB, together with AcrA and TolC.^44^ As expected, the MIC of cloxacillin was reduced about 35-fold in *acrB* mutant cells (DL5916) compared to wild type (*acrB*^*+*^) (DL5850) (Fig. 3e). However, addition of 0.5 × MIC of COE2-2hexyl reduced the MIC of cloxacillin similarly in *acrB*^*+*^ and *acrB* mutant cells (2-fold and 4-fold, respectively). Thus, COE2-2hexyl sensitizes cells to cloxacillin via an AcrB-independent pathway. Microscopic analysis indicated that 0.5 × MIC blocked cell septation based on the presence of long filaments after 3 h growth (Fig. 3f) and also inhibited cell motility based on microscopic observation. Together, these results suggest that COE2-2hexyl affects membrane-associated functions, which play essential roles in cell division and motility. We observed only single, mostly non-motile cells with no filaments at 2 × MIC, likely because cells did not grow based on the analysis of total protein content over the time course (Fig. 3d). Taken together, these data suggest that the loss of viability caused by COE2-2hexyl is not solely due to overt disruption of membrane integrity, but likely involves additional effects on several membrane-dependent processes as evidenced by alterations in cell septation, motility, ATP, respiration and cloxacillin susceptibility.

### Mechanistic studies of COE resistance

The evolution of bacterial resistance to COE2-2hexyl was analysed in *S*. Typhimurium and MRSA, using either serial dilution or morbidostat-based experimental evolution (see Methods). Serial dilution. No high-level COE^R^ mutants (≥50 × MIC) of either *S*. Typhimurium 14028 or CA-MRSA USA300 were isolated after serial dilution for 21 days; however, low-level COE^R^ mutants were recovered in both organisms (8 × MIC and 4 × MIC, respectively) (Supplementary Fig S1a; Supplementary Table S4a and b). Morbidostat. Although high-level CIP^R^ mutants of wild-type *E. coli* (BW25113) were obtained after morbidostat culture for >3 days (64-128 × MIC),^35^ no COE^R^ mutants were observed under these conditions. Therefore, *E. coli mutL* (JW4128) and wild-type *A. baumannii* ATCC 17978 were assessed for COE^R^ mutants since both strains possess an inherently higher frequency of mutation (∼10-100-fold), resulting from deficiencies in either methyl-directed mismatch repair or the intrinsic DNA damage-inducible response, respectively.^45^^,^^46^ Only low-level COE^R^ mutants (2-4 × MIC) were recovered from either of these hypermutable strains, which is in marked contrast with the high-level CIP^R^ mutants (128 × MIC) observed for *A. baumannii*^35^ (Supplementary Fig. S1b; Supplementary Table S4c–e).

Whole genome sequencing of COE^R^ mutants revealed mutation(s) in genes encoding essential functions involved in membrane remodelling and secretion (Supplementary Table S4a–e). Examples include: Gram-negative *secA* and *bamA* involved in outer membrane protein biogenesis and *lptD* from the lipopolysaccharide translocation complex^47^^,^^48^; *pgsA* from the phosphatidylglycerol biogenesis pathway^49^; and Gram-positive *pmtR* from the cytolytic peptide toxin transporter pathway.^50^ Consistent with this observation, a *de novo* constructed *S*. Typhimurium *secA* nonsense mutant (G880∗ (GGA→TGA))—derived from COE^R^ mutant analysis from serial-dilution—showed a two-fold increase in COE resistance (Supplementary Table S4a). Taken together, these data suggest that COE2-2hexyl did not evoke significant bacterial resistance, potentially due to specific effects on essential functions involved in membrane remodelling and secretion.

## Discussion

There is an increasing demand for new antibiotics that effectively treat patients with refractory bacteremia, do not evoke bacterial resistance, and can be readily modified to address current and anticipated patient needs. Here, we describe a promising compound, COE2-2hexyl, that exhibited broad-spectrum antibacterial activity. COE2-2hexyl effectively-treated mice infected with bacteria derived from sepsis patients with refractory bacteremia, including a CRE *K. pneumoniae* strain resistant to nearly all clinical antibiotics tested. Notably, this lead compound did not evoke drug resistance in several pathogens tested. COE2-2hexyl has specific effects on multiple membrane-associated functions (e.g., septation, motility, ATP synthesis, respiration, membrane permeability to small molecules) that may act together to abrogate bacterial cell viability and the evolution of drug-resistance. Impeding these bacterial properties may occur through alteration of vital protein–protein or protein-lipid membrane interfaces – a mechanism of action distinct from many membrane disrupting antimicrobials or detergents that destabilize membranes to induce bacterial cell lysis. The diversity and ease of COE design and chemical synthesis have the potential to establish a new standard for drug design and personalized antibiotic treatment.

COE2-2hexyl appears to differentially affect bacterial cell membranes, depending on COE concentration. At a sub-MIC level of COE2-2hexyl, cell septation, ATP level, susceptibility to cloxacillin, and motility were significantly affected along with a 3-fold decrease in viability, without altering membrane permeability or respiration. In contrast, at 2 × MIC, ∼50% of the cell population showed increased membrane permeability to small molecules accompanied by a 2 to 3-fold decrease in ATP levels and respiration, whereas viability was decreased ∼1000-fold. Thus, the marked effects of COE2-2hexyl on cell viability cannot be solely attributed to any individual membrane-based metabolic function tested. However, the COE-2-2hexyl-mediated disruption of multiple membrane-associated functions may act together in a synergistic manner to produce a larger effect on viability, which could occur by alteration of critical protein–protein or protein-lipid membrane interfaces. Consistent with this hypothesis, COE exposure resulted in morphological, mechanical, and compositional changes of the outer membrane of *E. coli*.^51^ In any case, the mechanism of action of COE2-2hexyl therefore appears to be distinct from the membrane-targeting antimicrobial colistin, which induces membrane destabilization and lysis.^52^ Notably, COE2-2hexyl is a quaternary ammonium compound (QAC), a chemical class of antimicrobial agents^53^ that has been shown to interact with the bacterial membrane^54^ and serve as topical agents^55^; however, their use for the treatment of systemic infections has been poorly studied.

COE2-2hexyl did not evoke significant resistance in Gram-negative and Gram-positive organisms by either serial dilution or morbidostat-based experimental evolution. These data suggest that the COE either targets membrane-associated functions encoded by essential genes or resistance results from alteration of membrane biochemical dynamics that inhibit essential membrane protein function(s). Furthermore, no mutations were observed that affect any of the numerous efflux pumps, which were readily obtained using the same approach for ciprofloxacin resistance,^35^ suggesting that COE2-2hexyl is not an efflux pump substrate or effector. MDR pathogens whose resistance is driven primarily by pre-existing efflux upregulation would thus remain susceptible to COEs. In this regard, COE2-2hexyl potentiated the effect of cloxacillin for *acrB*^*-*^ *E. coli* four-fold, which could occur by increasing membranes permeability. Notably, construction of a *S*. Typhimurium COE^R^ mutant in *secA* imparted a two-fold increase in MIC, suggesting that drug resistance is exerted, at least in part, through the Sec/Bam outer membrane protein biogenesis pathway of Gram-negative bacteria.^47^^,^^48^ Consistent with this observation, a similar *E. coli secA* nonsense mutation was shown to modify protein translocation via reduced binding to the SecB chaperone, which maintains precursor proteins in a translocation-competent state and targets them to SecA in the cytoplasmic membrane.^56^ Drug targeting of these essential membrane-associated functions also potentially contributes to the low frequency of resistance. This possibility is intriguing, given that membrane-disrupting agents are often selective for either Gram-negative or Gram-positive bacteria.^13^^,^^57^^,^^58^ Indeed, 14 of the 15 COEs tested were selective for Gram-positive bacteria. However, COE2-2hexyl, and its close synthetic derivatives, COE2-2pentyl and COE2-2heptyl, conferred broad-spectrum efficacy, raising the possibility that these COE derivatives alter both inner- and outer-membrane functions.

Recent studies have shown that small molecules can preferentially target bacterial membranes due to significant differences in lipid composition, presence of a cell wall, and the absence of cholesterol.^59^^,^^60^ The inner membranes of Gram-negative bacteria are generally more negatively charged at their surface because they contain more anionic lipids such as cardiolipin and phosphatidylglycerol^61^ within their outer leaflet compared to mammalian membranes.^62^^,^^63^ In contrast, membranes of mammalian cells are largely composed of more-neutral phospholipids, sphingomyelins, as well as cholesterol, which affords membrane rigidity and ability to withstand mechanical stresses^64^; and may stabilize the membrane against structural damage to membrane-disrupting agents such as COEs. Consistent with these studies, COE2-2hexyl was well tolerated in mice, suggesting that COEs are not intrinsically toxic *in vivo*, which is often a primary concern with membrane-targeting antibiotics.

The ease of molecular design and modular nature of COEs offer many advantages over conventional antimicrobials due to their intermediate molecular size, sufficient aqueous solubility to achieve efficacy, and the absence of complex chemical structures/chiral centers, making synthesis simple, scalable and affordable. The COE refinement workflow potentially accelerates lead-compound optimization by more rapid screening of novel compounds for the iterative directed-design process. It also reduces the time and cost of subsequent biophysical characterization, medicinal chemistry and bioassays, ultimately facilitating the discovery of novel compounds with improved pharmacological properties. Additionally, COEs provide an approach to gain new insights into microbial physiology, including membrane structure/function and mechanism of drug action/resistance, while also generating a suite of tools that enable the modulation of bacterial and mammalian membranes for scientific or manufacturing uses. Notably, further COE safety and efficacy studies will need to be conducted on a larger scale to ensure adequate understanding of the clinical benefits and risks to assure clinical efficacy and toxicity before COEs can be added to the therapeutic armamentarium. Despite these limitations, the modular design of COEs enables the construction of a spectrum of compounds with the potential as a new versatile therapy for the emergence and rapid global spread of pathogens that are resistant to all, or nearly all, existing antimicrobial medicines.

## Contributors

Experiments were conducted by D.M.H., S.P.M., L.B., S.A.L, C.X.G., J.E.Z., J.L. and D.A.L. Data were analysed by D.M.H., S.P.M., L.B., S.A.L., C.X.G., J.E.Z, G.C.B., J.C.F, L.N.F, J.K.H., C.E.S., A.L.O, D.A.L., and M.J.M. The manuscript was prepared by D.M.H., S.P.M., C.E.S, A.L.O., D.A.L., and M.J.M. The study was planned and directed by D.M.H., S.P.M, G.C.B., A.L.O., D.A.L., and M.J.M. All authors read and approved the final manuscript. M.J.M is the guarantor of this work. Both D.M.H and M.J.M have verified the underlying data of this manuscript.

## Data sharing statement

All data generated or analysed during this study are included in this published article or in the supplement.

## Declaration of interests

A UC Santa Barbara patent application describing the composition and use of COEs and derivatives as antibiotics is currently pending (G.C.B., M.J.M., D.M.H., J.L.; US20210017179A1). G.C.B. is a principal of Xiretsa Inc., which had no role in the design, execution, analysis, or funding of the work. The remaining authors made no declarations.

## Appendix A Supplementary data

Suppl TOC, Fig. S1, Tables S1–S3

Supplementary Table S4.xlsx

Caption of Supplementary Material
