# Supplementary material for: A broad-spectrum synthetic antibiotic that does not evoke bacterial resistance
Source: eBioMedicine. 2023 Feb 15;89:104461. doi: 10.1016/j.ebiom.2023.104461 (PMC10025758; doi:10.1016/j.ebiom.2023.104461)
Supplement: Suppl TOC, Fig. S1, Tables S1–S3 [file mmc1.docx]

**Supplementary Online Content**

**Heithoff, DM, Mahan, SP, Barnes, L et al. “*A Broad-spectrum Synthetic Antibiotic That Does Not Evoke Bacterial Resistance*.” *EbioMedicine.***

1. Table of Contents.................................................................................................................................page 1

2. Supplementary Figure 1. Evolution of bacterial resistance to COE2-2hexyl.......................................page 2

3. Supplementary Table 1. COE structure, antibacterial activity, and cytotoxicity.................................page 3

4. Supplementary Table 2. AST determined on *K. pneumoniae* and MRSA isolates derived from

sepsis patients refractory to antibiotic treatment...............................................................................page 9

5. Supplementary Table 3. Antibacterial activity of COE2-2hexyl derivatives against clinical

bacterial isolates...............................................................................................................................page 10

6. Supplementary References...................................................................................................................page 11

7. Supplementary Table 4. COE resistant mutants....................................................................... Supplementary data set

Supplementary Table 4 Table of Contents

Supplementary Table 4a. *S*. Typhimurium (serial dilution)

Supplementary Table 4b. *S. aureus* (serial dilution)

Supplementary Table 4c. *E. coli mutL* (morbidostat)

Supplementary Table 4d. *A. baumannii* population (morbidostat)

Supplementary Table 4e. *A. baumannii* single colony isolates (morbidostat)


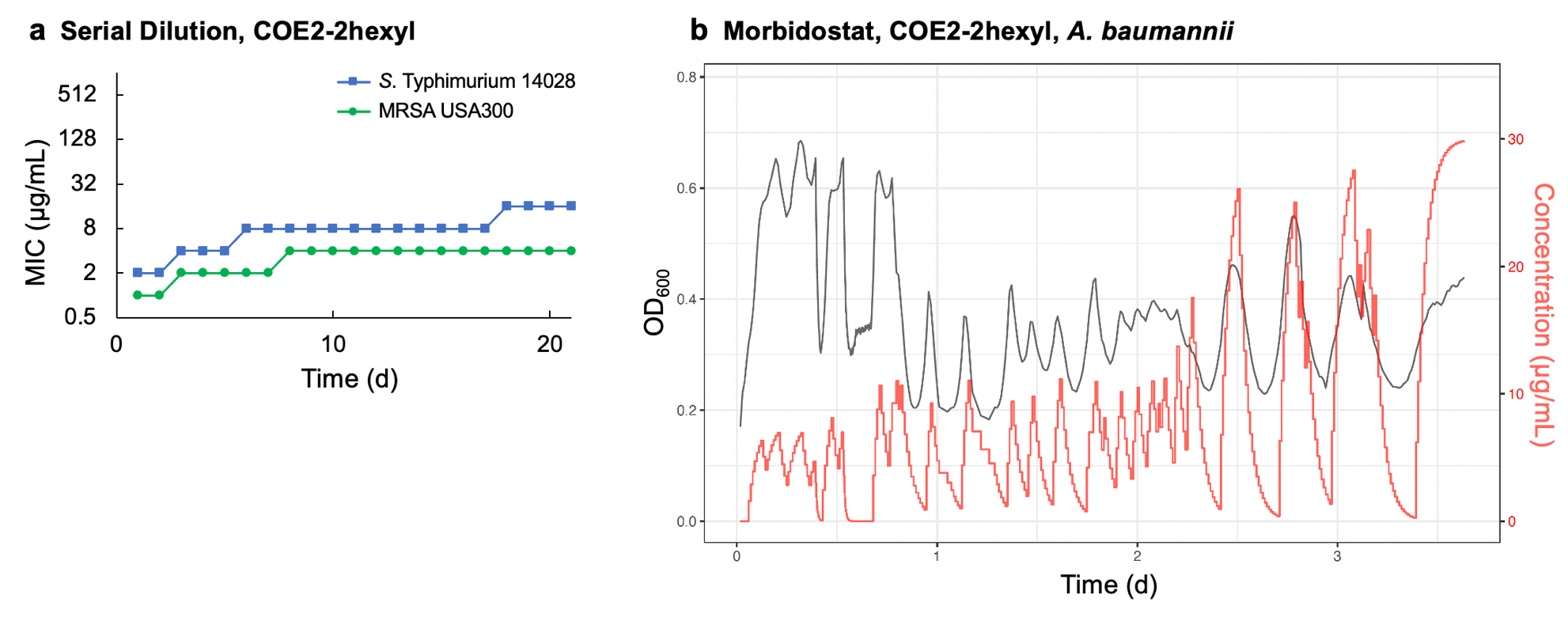


**Supplementary Figure 1.** **Evolution of bacterial resistance to COE2-2hexyl.** **a**, Serial dilution. Bacteria were grown overnight, serially diluted 1:10 in the presence of 1X MIC COE2-2hexyl and incubated 20 h at 37^o^C. MICs for *S.* Typhimurium 14028 and CA-MRSA USA300 are 2 µg/mL and 1 µg/mL, respectively. If growth occurred, cells were challenged at 2X MIC; and this procedure was repeated for 21 consecutive days. Values given are the consensus MIC for 3 independent lineages. **b**, Morbidostat. *A. baumannii* ATCC 17978 was grown in a morbidostat for > 3 days in the presence of COE2-2hexyl at the indicated cell density (OD_600_, black line) and drug concentration (red line). The *A. baumannii* culture responded to ~20 µg/mL COE2-2hexyl (2.5X MIC) with recovery growth at 0.5X MIC (4 µg/mL). WT MIC = 8 µg/mL.

| **Supplementary Table 1. COE structure, antibacterial activity, and cytotoxicity.** | | | | | | | | | | |  |  |
| --- | --- | --- | --- | --- | --- | --- | --- | --- | --- | --- | --- | --- |
|  | | Antibacterial activity | |  |  |  | Structural Attributes | | | | |  |
| COE Structure | | MIC (μg/mL) | |  | Cytotoxicity |  | Terminal  end group | Linkage  type | Pendant length | Core  rings | | |
|  |  | Gram‒ | Gram+ |  | (% viability) |  |  |  |  |  |  |  |
| *Terminal end groups* | |  |  |  |  |  |  |  |  |  | | |
| 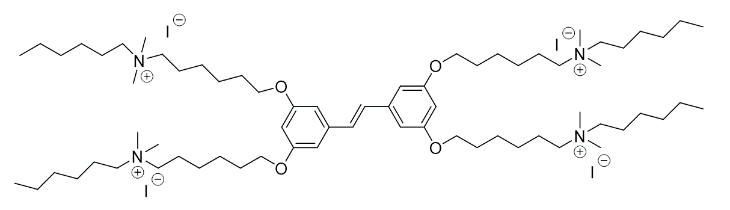COE2-2hexyl | 2‒8 | | 1 |  | 98.5% |  | trimethyl-hexyl-ammonium  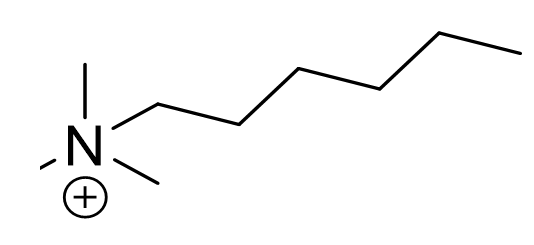 | ether | 6 | 2 | | |
| COE2-2C   | 32‒256 | | 4 |  | 86% |  | tetramethylammonium  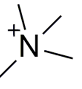 | ether | 6 | 2 | | |
| COE2-2py   | 8‒128 | | 0.5‒2 |  | 91% |  | pyridinium  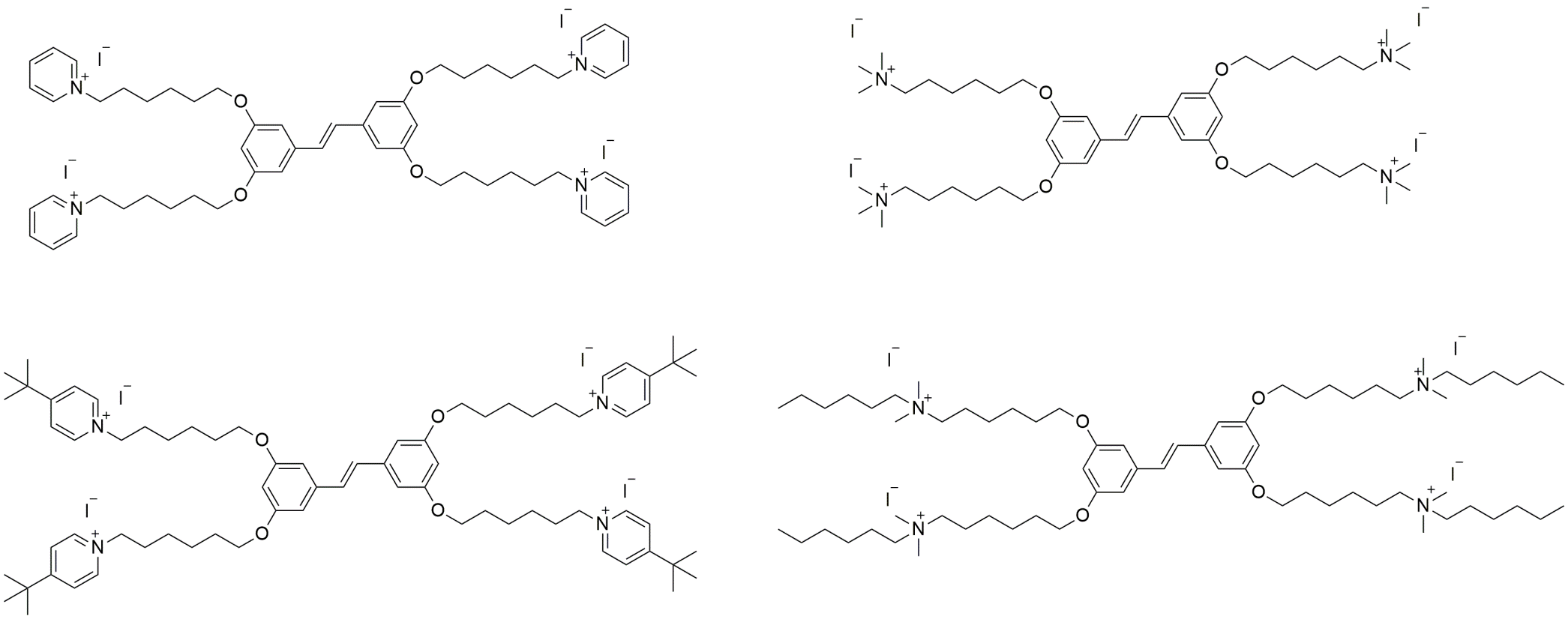 | ether | 6 | 2 | | |
| COE2-2B | >16 | | >16 |  | 87% |  | tert-butyl-methylpyridinium  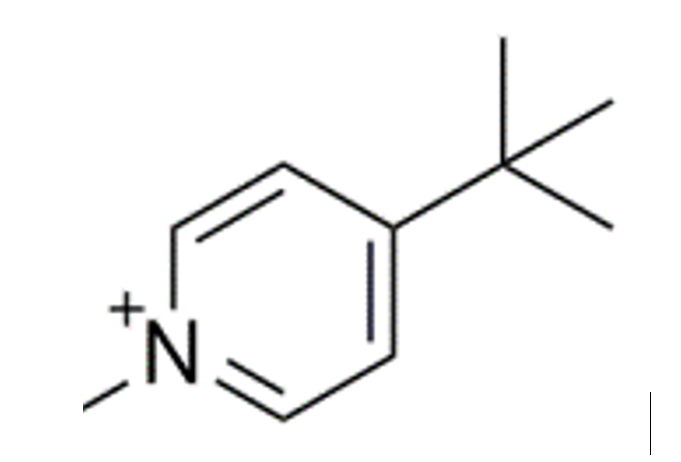 | ether | 6 | 2 | | |

| *Core repeat number* |  |  |  |  |  |  |  |  |  |
| --- | --- | --- | --- | --- | --- | --- | --- | --- | --- |
| COE2-2C   | 32‒256 | 4 |  | 86% |  | tetramethylammonium  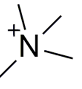 | ether | 6 | 2 |
| COE2-3C   | 8‒256 | 0.5‒1 |  | 93% |  | tetramethylammonium  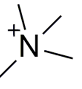 | ether | 6 | 3 |
| 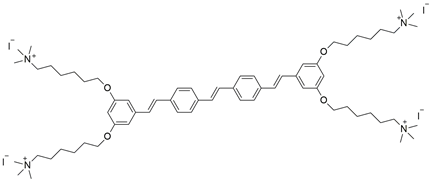COE2-4C | >128 | 1‒2 |  | <1% |  | tetramethylammonium  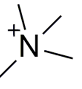 | ether | 6 | 4 |
| COE2-3-pCp   | 32‒>128 | ≤0.25‒0.5 |  | 40% |  | tetramethylammonium  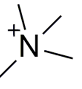 | ether | 6 | 4 |

| *Pendant length* |  |  |  |  |  |  |  |  |  |
| --- | --- | --- | --- | --- | --- | --- | --- | --- | --- |
| COE2-3C-C4   | 4‒256 | 2‒4 |  | 91% |  | tetramethylammonium  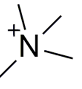 | ether | 4 | 3 |
| COE2-3C | 8‒256 | 0.5‒1 |  | 93% |  | tetramethylammonium  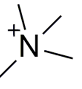 | ether | 6 | 3 |
| COE2-2C | 32‒256 | 4 |  | 86% |  | tetramethylammonium  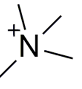 | ether | 6 | 2 |
| COE2-2C-C8 | 4‒64 | 0.5 |  | 87% |  | tetramethylammonium  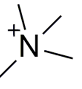 | ether | 8 | 2 |

| *End group PEGylation* |  |  |  |  |  |  |  |  |  |
| --- | --- | --- | --- | --- | --- | --- | --- | --- | --- |
| 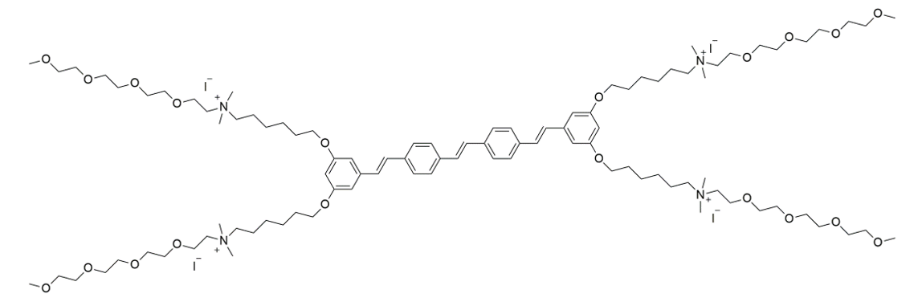COE2-4PC | >128 | 4‒8 |  | 94% |  | PEGylated-TMA  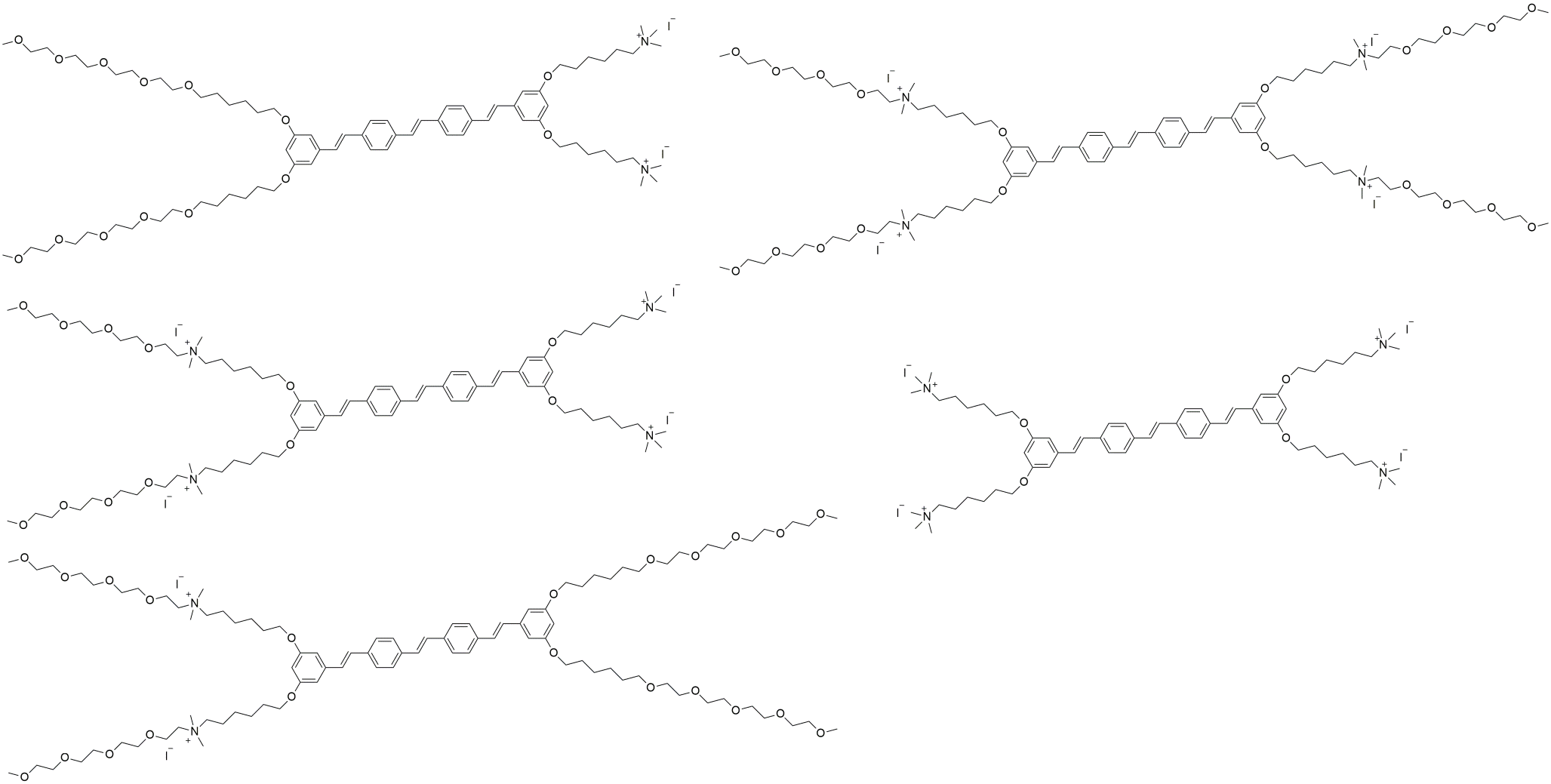 | ether | 6 | 4 |
| COE2-4P:C | >128 | >128 |  | 18% |  | TMA (2/4) + PEG (2/4)  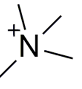   | ether | 6 | 4 |
| COE2-4PC:C   | >128 | 4 |  | 28% |  | TMA (2/4) +  PEGylated-TMA (2/4)  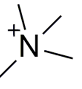 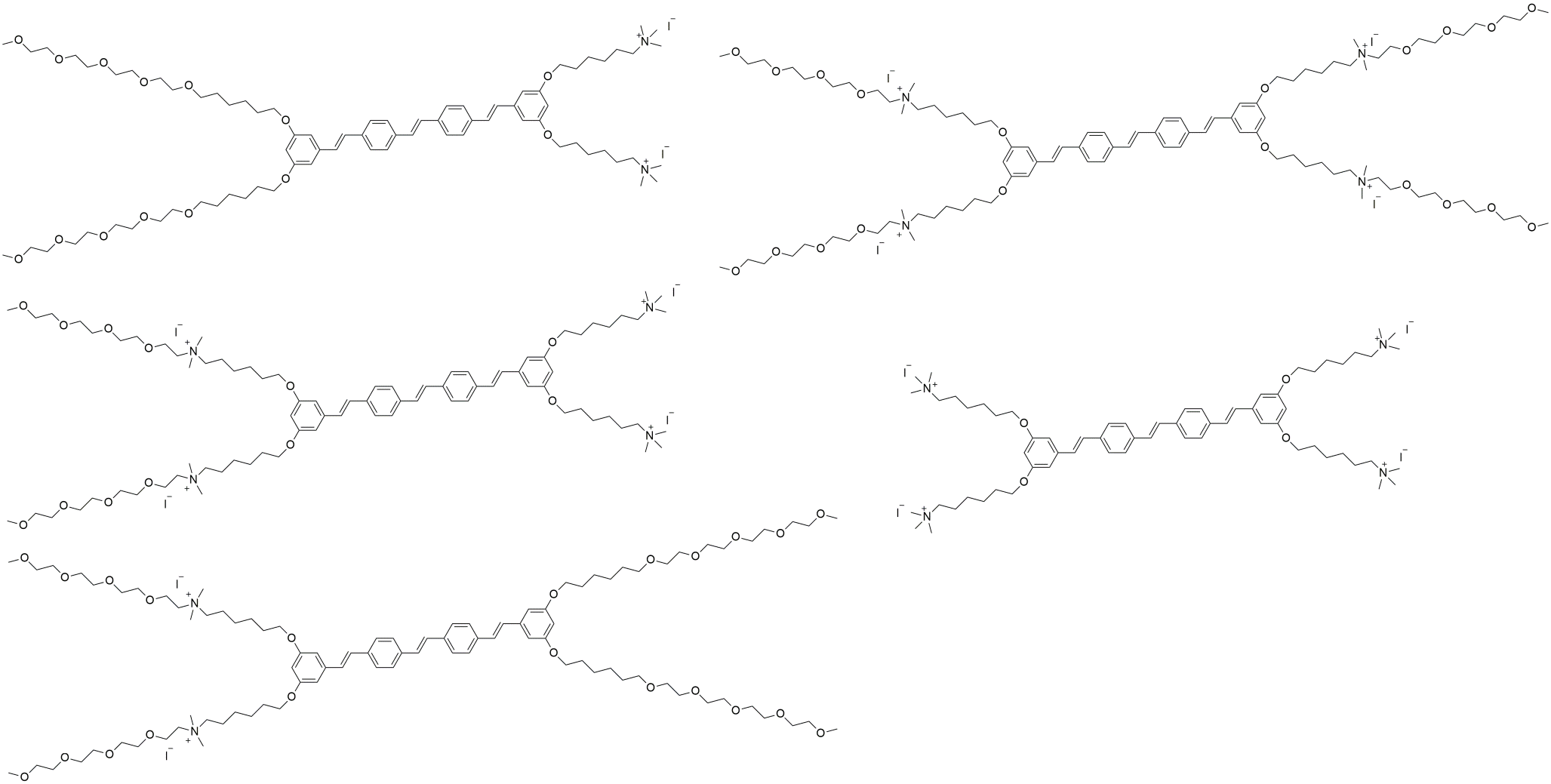 | ether | 6 | 4 |
| COE2-4PC:P  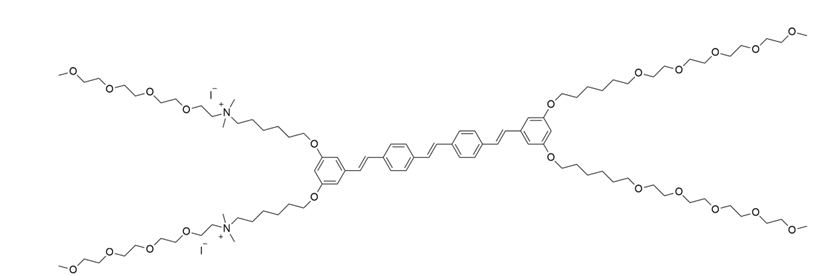 | >128 | >128 |  | 70% |  | PEGylated-TMA (2/4) +  PEG (2/4)  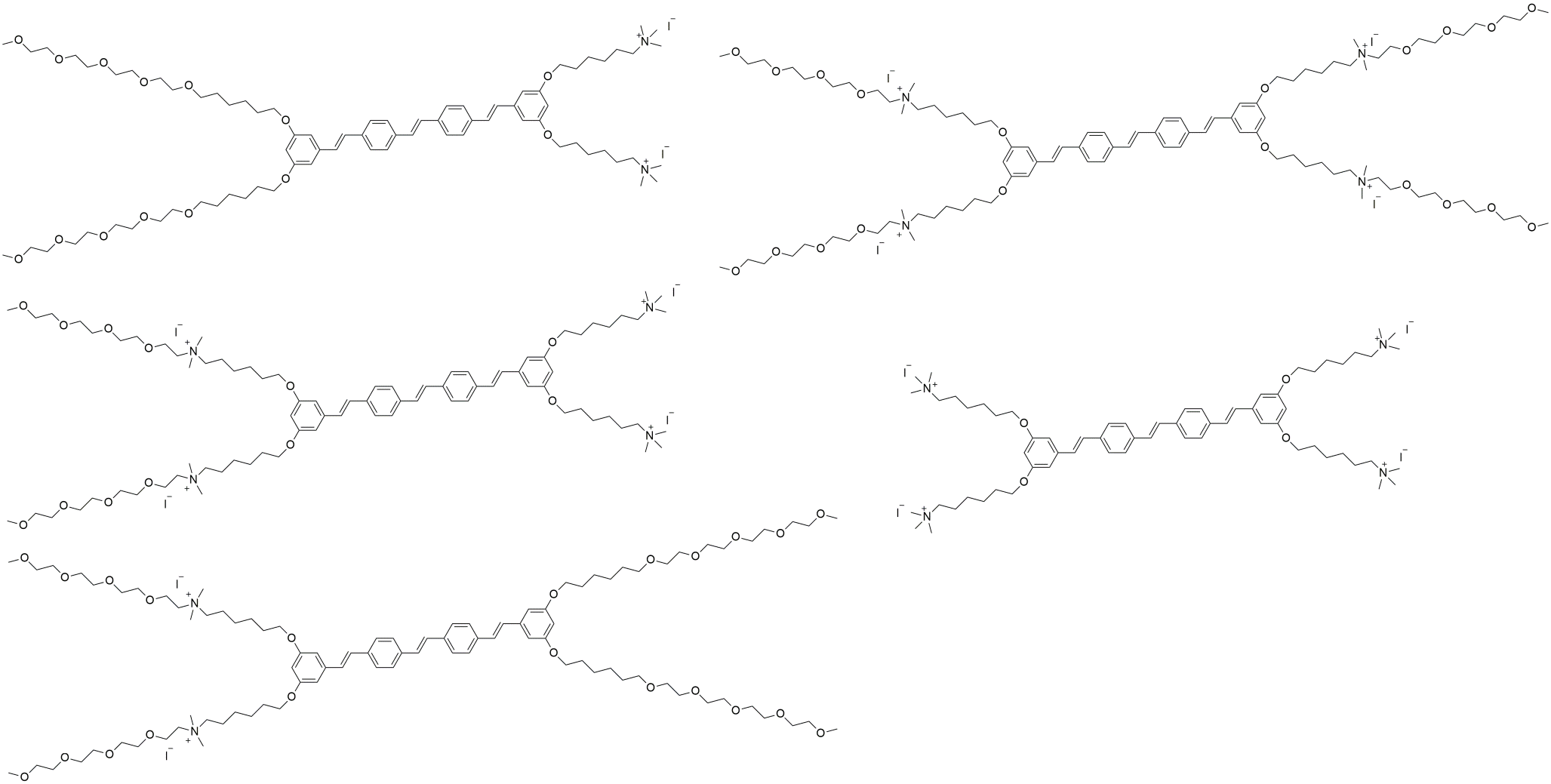  | ether | 6 | 4 |

| COE2-4C  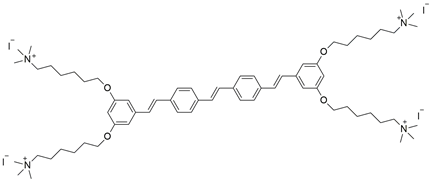 | >128 | 1‒2 |  | <1% |  | tetramethylammonium (4/4)  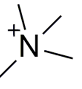 | ether | 6 | 4 |
| --- | --- | --- | --- | --- | --- | --- | --- | --- | --- |
| *Alkyl-chain linkage type* |  |  |  |  |  |  |  |  |  |
| DSBN   | 64‒ >256 | 1‒2 |  | 98% |  | tetramethylammonium  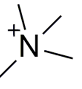 | tertiary amine | 6 | 3 |
| COE2-3C   | 8‒256 | 0.5‒1 |  | 93% |  | tetramethylammonium  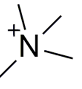 | ether | 6 | 3 |
| DSSN   | >256 | 2 |  | 39% |  | tetramethylammonium  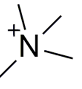 | tertiary amine | 6 | 4 |

| 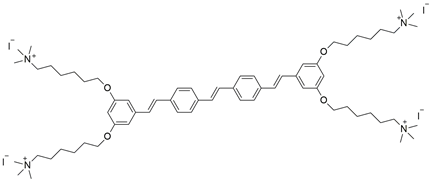COE2-4C | >128 | 1‒2 |  | <1% |  | tetramethylammonium  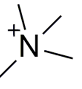 | ether | 6 | 4 | |
| --- | --- | --- | --- | --- | --- | --- | --- | --- | --- | --- |
| COE syntheses and product characterizations were as previously described (detailed experimental methods, synthetic schemes, yields and NMR spectra).^1-5^ COE structural variants (depicted) were evaluated for antibacterial activity and cytotoxicity. Antibacterial activity: MICs were determined against a collection of 9 clinical bacterial isolates via broth microdilution^6-8^ (n > 9) (see Methods). Cytotoxicity: COE structural variants (4 μg/mL) were assessed for *in vitro* cytotoxicity relative to ciprofloxacin (2 μg/mL) in a murine macrophage cell line (RAW 264.7) determined by the Trypan blue dye exclusion method^9^ (n = 6) (see Methods). Values given are % viability after COE treatment; % viability after ciprofloxacin treatment = 94%. Tetramethylammonium (TMA). | | | | | | | | | |  |

| **Supplementary Table 2. AST determined on *K. pneumoniae* and MRSA isolates derived from sepsis patients refractory to antibiotic treatment.** | | | | | | |
| --- | --- | --- | --- | --- | --- | --- |
| Antibiotic | CRE *K. pneumoniae*  MT3325 | | |  | MRSA blood isolate  MT3302 | |
|  | MIC (μg/mL) |  |  | | MIC (μg/mL) |  |
| Amoxicillin/Clavulanate | > 16/8* | R |  | | - | - |
| Ampicillin | > 512** | R |  | | 256 | R |
| Ampicillin/Sulbactam | ≥ 32* | R |  | | - | - |
| Azithromycin | 128 | R |  | | 128 | R |
| Aztreonam | > 16* | R |  | | - | - |
| Cefazolin | ≥ 64* | R |  | | - | - |
| Cefepime | ≥ 64* | R |  | | - | - |
| Cefotaxime | > 32* | R |  | | - | - |
| Ceftazidime | ≥ 64* | R |  | | - | - |
| Ceftiofur | ≥ 512 | R |  | | 16 | R |
| Ceftriaxone | > 512** | R |  | | 128 | R |
| Cefuroxime | > 16* | R |  | | - | - |
| Cephalexin | > 512 | R |  | | 128 | R |
| Cephalothin | - | - |  | | 8 | S |
| Chloramphenicol | ≥ 512 | R |  | | 8 | S |
| Ciprofloxacin | 128** | R |  | | 0.25** | S |
| Clindamycin | - | - |  | | 0.25** | S |
| Colistin Sulfate | 0.125 | S |  | | - | - |
| Daptomycin | - | - |  | | 0.5** | S |
| Doxycycline | - | - |  | | ≤ 0.5* | S |
| Enrofloxacin | ≥ 16 | R |  | | 0.25 | - |
| Ertapenem | 256** | R |  | | 4 | I |
| Erythromycin | ≥ 512 | - |  | | 128** | R |
| Florfenicol | ≥ 32 | - |  | | 4 | - |
| Gentamicin | 4** | S |  | | 1** | S |
| Imipenem | 32** | R |  | | 0.125 | S |
| Kanamycin | 64 | R |  | | > 512 | R |
| Levofloxacin | ≥ 8* | R |  | | 0.25* | S |
| Linezolid | - | - |  | | 2** | S |
| Moxifloxacin | - | - |  | | ≤ 0.25* | S |
| Nalidixic Acid | ≥ 512 | R |  | | 64 | - |
| Neomycin | 1 | S |  | | - | - |
| Nitrofurantoin | 256* | R |  | | 32* | S |
| Oxacillin | - | - |  | | 64** | R |
| Piperacillin/Tazobactam | > 512/4** | R |  | | 64/4 | R |
| Polymyxin B | 0.25 | S |  | | - | - |
| Rifampin | - | - |  | | 0.008** | S |
| Spectinomycin | ≥ 512 | R |  | | 128 | - |
| Streptomycin | 128 | R |  | | 8 | S |
| Tetracycline | 4** | S |  | | 0.5** | S |
| Ticarcillin/Clavulanate | > 64* | R |  | | - | - |
| Tigecycline | - | - |  | | ≤ 0.12* | S |
| Tobramycin | ≥ 16* | R |  | | - | - |
| Trimethoprim | ≥ 128** | R |  | | 2 | S |
| Co-Trimoxazole | > 32/608** | R |  | | 0.125/2.4** | S |
| Vancomycin | - | - |  | | 1** | S |
| MICs and clinical breakpoints and were determined by either broth microdilution^6-8^ (designated by no asterisk; n = 3); or by VITEK (bioMerieux, Inc.; designated by *); or by both broth microdilution and VITEK (designated by **). S = Susceptible, I = Intermediate, R = Resistant. | | | | | | |

| **Supplementary Table 3. Antibacterial activity of COE2-2hexyl derivatives against clinical bacterial isolates.** | | | | |
| --- | --- | --- | --- | --- |
|  | Antibacterial activity | | | |
|  | MIC (μg/mL) | | | |
| Pathogen | | COE2-2pentyl | COE2-2hexyl | COE2-2heptyl |
| *Gram-negative* | |  |  |  |
| *E. coli* ATCC 25922 | | 2 | 2 | 2 |
| *K. pneumoniae* ATCC 13883 | | 4 | 2 | 2 |
| *P. aeruginosa* ATCC 10145 | | 16 | 8 | 8 |
| *S.* Typhimurium 14028 | | 2 | 2 | 2 |
| *Gram-positive* | |  |  |  |
| MSSA Newman | | 0.5 | 1 | 1 |
| MSSA blood isolate (MT3305) | | 0.5 | 1 | 1 |
| CA-MRSA USA300 | | 0.5 | 1 | 1 |
| MRSA blood isolate* (MT3302) | | 0.5 | 1 | 1 |
| MRSA wound isolate (MT3315) | | 0.5 | 1 | 1 |
| MICs were determined by broth microdilution^6-8^ (n > 9). | | | | |

**Supplementary References**

1. Limwongyut J, Nie C, Moreland A, Bazan G. Molecular design of antimicrobial conjugated oligoelectrolytes with enhanced selectivity toward bacterial cells. *Chem Sci* 2020; **11**(31): 8138-44.

2. Yan H, Rengert Z, Thomas A, Rehermann C, Hinks J, Bazan G. Influence of molecular structure on the antimicrobial function of phenylenevinylene conjugated oligoelectrolytes. *Chem Sci* 2016; **7**(9): 5714-22.

3. Zhou C, Chia G, Ho J, et al. Informed molecular design of conjugated oligoelectrolytes to increase cell affinity and antimicrobial activity. *Angew Chem* 2018; **130**(27): 8201-4.

4. Tiihonen A, Cox-Vazquez S, Liang Q, et al. Predicting antimicrobial activity of conjugated oligoelectrolyte molecules via machine learning. *J Am Chem Soc* 2021; **143**(45): 18917-31.

5. Bazan G, Zhang Z, Moreland A, et al. Short conjugated oligoelectrolytes and uses thereof. 2021. <https://patents.google.com/patent/US20210017179A1/en> (accessed November 30, 2022).

6. Clinical and Laboratory Standards Institute. Performance standards for antimicrobial resistance testing; twenty-fourth informational supplement. 2014.

7. Clinical and Laboratory Standards Institute. Performance Standards for Antimicrobial Susceptibility Testing, 31st Edition 2021.

8. European Committee on Antimicrobial Susceptibility Testing. Breakpoint tables for interpretation of MICs and zone diameters. Version 6.0. 2016.

9. Kamiloglu S, Sari G, Ozdal T, Capanoglu E. Guidelines for cell viability assays. *Food Frontiers* 2020; **1**(3): 332-49.
