# Supplementary material for: A broad-spectrum synthetic antibiotic that does not evoke bacterial resistance
Source: eBioMedicine. 2023 Feb 15;89:104461. doi: 10.1016/j.ebiom.2023.104461 (PMC10025758; doi:10.1016/j.ebiom.2023.104461)
Supplement: Caption of Supplementary Material [file mmc3.docx]

**Caption for Supplementary Material**

**Heithoff, DM, Mahan, SP, Barnes, L et al. “*A Broad-spectrum Synthetic Antibiotic That Does Not Evoke Bacterial Resistance*.” *EbioMedicine.***

Supplementary Figure 1. Evolution of bacterial resistance to COE2-2hexyl

Supplementary Table 1. COE structure, antibacterial activity and cytotoxicity

Supplementary Table 2. AST determined on *K. pneumoniae* and MRSA isolates derived from sepsis patients refractory to antibiotic treatment

Supplementary Table 3. Antibacterial activity of COE2-2hexyl derivatives against clinical bacterial isolates

Supplementary References

Supplementary Table 4. COE resistant mutants
